# Dynamic Diffusion Tensor Imaging Reveals Structural Changes in the Bilateral Pyramidal Tracts after Brain Stem Hemorrhage in Rats

**DOI:** 10.3389/fnana.2016.00033

**Published:** 2016-03-30

**Authors:** Ru-Zhi Zhang, Chuan-Yuan Tao, Wei Chen, Chun-Hua Wang, Yue Hu, Li Song, Bing Zhang, Yu-Shu Chen, Zi-Qian Xu, Lei Wang, Hua Feng, Ting-Hua Wang, Jie Zheng, Chao You, Fa-Bao Gao

**Affiliations:** ^1^Department of Radiology, West China Hospital, Sichuan UniversityChengdu, China; ^2^Department of Neurosurgery, West China Hospital, Sichuan UniversityChengdu, China; ^3^Department of Anesthesiology and Institute of Neurological Disease, Translation Neuroscience Center, West China Hospital, Sichuan UniversityChengdu, China; ^4^Department of Neurosurgery, Southwest Hospital, Third Military Medical UniversityChongqing, China; ^5^Mallinckrodt Institute of Radiology, Washington University School of MedicineSt. Louis, MO, USA

**Keywords:** brain stem hemorrhage, diffusion tensor imaging, pyramidal tract, demyelination, myelin repair

## Abstract

**Background and Purpose**: Few studies have concentrated on pyramidal tract (PY) changes after brain stem hemorrhage (BSH). In this study, we used a diffusion tensor imaging (DTI) technique and histologic identification to investigate longitudinal PY changes on both the contralateral and ipsilateral sides after experimental BSH.

**Methods**: BSH was induced in 61 Sprague-Dawley rats by infusing 30 μl of autogenous tail blood into each rat’s right pons. DTI and motor function examinations were performed repeatedly on days 1, 3, 7, 14, and 28 after surgery. Fractional anisotropy (FA), mean diffusivity (MD), axial diffusivity, and radial diffusivity were measured in the bilateral PYs. The axon and myelin injury in the PY were evaluated by histologic study.

**Results**: As compared with normal controls, the bilateral PYs in rats with induced BSH showed an early decrease and a late increase in FA and an early increase and a late decrease in MD. A progressive decrease in axial diffusivity with dramatic axon loss from day 1 to day 28 after BSH was found bilaterally. The bilateral PYs showed an early increase and a late decrease in radial diffusivity. Early myelin injury and late repair were also detected pathologically in the bilateral PYs of rats with BSH. Thus, the early motor function deficits of rats with BSH began to improve on day 14 and had almost completely disappeared by day 28.

**Conclusions**: DTI revealed dynamic changes in the bilateral PYs after BSH, which was confirmed by histologic findings and which correlated with motor function alteration. These findings support the idea that quantitative DTI can track structural changes in the bilateral PYs and that DTI may serve as a noninvasive tool to predict the prognoses of patients with BSH.

## Introduction

Primary brain stem hemorrhage (BSH), as one subtype of intracerebral hemorrhage (ICH; Qureshi et al., 2001; Xi et al., 2006), has a notoriously poor prognosis and an overall mortality rate of 65% despite contemporary medical-surgical approaches (Wijdicks and St Louis, 1997; Lekic et al., 2013; Meguro et al., 2015). Moreover, for those patients who survive BSH, the long-term disability rate is dramatically high, mainly as a result of injury to the pyramidal tract (PY; Takeuchi et al., 2013). The PY is the most important part of the motor pathway and one of the most important neural tracts for motor function in the human brain (Jang, 2011). Its injury can lead to motor deficits, and its recovery is mandatory for normal motor functioning. However, little is known about the structural changes that occur in the bilateral PYs after BSH.

Hematoma-induced secondary injury of the motor pathway has been increasingly investigated with the use of diffusion tensor imaging (DTI; Fan et al., 2013). Fractional anisotropy (FA) and mean diffusivity (MD) are commonly used indices that reflect white matter injury and neurologic deficit in individuals with ICH (Yoshioka et al., 2008; Kuzu et al., 2012; Fan et al., 2013). The greatest advantage of FA for the evaluation of white matter injury is that it comprehensively reflects the integrity of the fiber tract. However, both FA and MD fail to reveal the detailed pathologic processes that underlie white matter injury, and they cannot discriminate between axonal damage and myelin injury (Alexander et al., 2007).

The parameter λ_1_, which represents water diffusivity parallel to the axonal fibers, is also referred to as the *axial diffusivity* (λ_∥_; Song et al., 2002). The parameters λ_2_ and λ_3_, which represent the water diffusivities perpendicular to the axonal fibers, are averaged and referred to as the *radial diffusivity* (λ_⊥_). Thus, a decrease in λ_∥_ is thought to be primarily related to axonal damage, whereas an increase in λ_⊥_ is thought to be related to myelin injury (Song et al., 2002; Kumar et al., 2011; van der Eerden et al., 2014). Both indices provide subtler pathologic information (i.e., axonal vs. myelin injury) than do other scalar DTI parameters, such as FA and MD (Kumar et al., 2011; van der Eerden et al., 2014).

During the past decade, the use of DTI has allowed great progress to be made regarding the discovery of the mechanisms of motor pathway injury and repair after ICH (Chaudhary et al., 2015). However, these previous DTI studies have mostly focused on supratentorial ICH; little is known about evolutionary injury and repair in the PY after BSH. The purpose of this study was to investigate the dynamic structural changes that occur in the bilateral PYs after experimental BSH with the use of DTI and histology.

## Materials and Methods

### BSH Model

Ninety-five male Sprague-Dawley rats (320 ± 20 g) were used in the experiment. Sixty-one rats were anesthetized with pentobarbital (50 mg/kg intraperitoneal) and injected with 30 μl of autogenous tail blood in the right pons (2 mm posterior and 1.3 mm lateral to the posterior fontanelle, 9.3 mm ventral to the dura). Twenty-six rats underwent the same operation following the same procedures without blood injection and were used as sham controls. Another eight rats were used as normal controls. All of the experimental procedures were approved by the local animal committee of West China Hospital, Sichuan University, Chengdu, China.

### Magnetic Resonance Imaging Methods

All of the magnetic resonance imaging experiments were performed with the use of a 7.0 T magnetic resonance scanner (Bruker Biospec 70/30, Ettlingen, Germany). All rats were anesthetized with a 2% isoflurane/oxygen mixture. Their body temperatures were kept constant at 37°C with the use of a heating blanket and monitored with a rectal temperature probe. T2- and T2*-weighted imaging were performed 2–4 h after blood injection to exclude rats with failed brain stem blood mimicking. BSH was successfully induced in 61 rats as evidenced by regularly shaped hematomas and no obvious blood reflux; four rats died immediately after blood injection. The surviving 57 rats with BSH were imaged on days 1 (D1; *n* = 57), 3 (D3; *n* = 25), 7 (D7; *n* = 15), 14 (D14; *n* = 9), and 28 (D28; *n* = 6) after surgery. Of the 57 rats with BSH, 33 rats died during the experiment, and 24 were sacrificed for histologic analysis. Twenty-six sham rats were imaged on D1 (*n* = 26), D3 (*n* = 21), D7 (*n* = 16), D14 (*n* = 11), and D28 (*n* = 6) after surgery. Eight normal controls were imaged at the age of 13 weeks, which corresponded with the average ages of the rats with BSH on D0 (13 weeks ± 1 week). The imaging protocol for all rats included a T2 spin-echo sequence (repetition time/echo time = 4000/33 ms); a T2* gradient-echo sequence (repetition time/echo time = 250/5 ms); and a spin-echo echo-planar DTI sequence with 30 diffusion gradient directions at *b* = 1000 s/mm^2^ and five additional images at *b* = 0 s/mm^2^ (repetition time/echo time = 4000/33 ms). The field of view (FOV) was 35 × 35 mm, and the matrix was 256 × 256 mm. Twenty-five coronal slices that were each 1 mm thick were acquired from each rat.

### Behavioral Tests

Three behavioral tests were performed: a forelimb placing test, a forelimb use asymmetry (cylinder) test, and a corner turn test. The advantages of these tests for the behavioral evaluation of rats have previously been validated (Hua et al., 2002). All behavioral tests were performed by a highly experienced experimenter who was blinded to the conditions of the animals before magnetic resonance scanning at each time point.

#### Measurement of Forelimb Placing

The first behavioral test was a vibrissae-elicited forelimb placing test to evaluate the ability of each rat to respond to vibrissae-elicited excitation as previously described (Hua et al., 2002). The animals were held by their trunks, positioned parallel to a countertop, and then slowly moved up and down to allow the vibrissae on one side of the head to brush along the countertop surface. Intact animals quickly placed their forelimbs ipsilateral to the stimulated vibrissae onto the countertop. Each rat was tested 10 times for each forelimb. The forelimb placing score was calculated as the number of successful forelimb placements on the edge of the countertop in response to the vibrissae stimulation out of the 10 consecutive trials.

#### Forelimb Use Asymmetry Test

The rats’ forelimb use during the exploratory activity was analyzed with the rat in a transparent cylinder (diameter, 20 cm; height, 30 cm). The behavior was scored by assessing each rat’s independent use of the left or right forelimb to contact the wall during a full rear to initiate a weight-shifting movement and the simultaneous use of both the left and right forelimbs to contact the wall (Hua et al., 2002). The behavior was quantified by determining the number of occasions when the ipsilateral (unimpaired) forelimb (I), the contralateral (impaired) forelimb (C), and both forelimbs (B) were used as a percentage of the total number of times that any limb contacted the wall. The forelimb use asymmetry score was calculated as follows: (I/[I + C + B]) − (C/[I + C + B]).

#### Corner Turn Test

The corner turn test was performed as previously described (Hua et al., 2002). Briefly, each rat was permitted to approach a 30° corner made out of two attached Plexiglas walls. To exit the corner, the rat had to turn either to the right or to the left. Only turns involving full rearing along either wall were included; ventral tucks and horizontal turns were excluded. The rats were not taken out of the cage immediately after each turn to avoid the development of an aversion to their prepotent turning responses. The choice of the turning side was recorded for 10 trials per test day, with an interval of at least 30 s placed between each trial. The corner turn score was calculated as follows: (Number of left turns/All turns) × 100%.

### Tissue Preparation

Brain samples from the rats with BSH (*n* = 7 on D1, *n* = 4 on D3, *n* = 4 on D7, *n* = 3 on D14, and *n* = 6 on D28) and from the sham controls (*n* = 5 on D1, D3, D7, and D14 and *n* = 6 on D28) were obtained immediately after magnetic resonance imaging. Five normal rats were also sacrificed after imaging for histologic analysis. Immediately after imaging, the rats were euthanized, and they then underwent fixation via transcardial perfusion with 4% paraformaldehyde in 1% phosphate-buffered saline (PBS). Coronal brain sections that were 2–3 mm thick and that contained PY tissue were excised, with the standard rat brain atlas used as reference (Paxinos and Watson, 2007). These sections were fixed overnight and then decalcified for 48 h; the fixed sections were embedded in paraffin. The sections that contained PY tissue were cut on a sliding microtome at a thickness of 5 μm.

### Immunohistochemistry

Sections were stained for myelin with the use of luxol fast blue stain (LFB; Abcam) and for axons using immunohistochemical staining for phosphorylated neurofilaments (SMI-31; Abcam; Budde et al., 2007). Immunohistochemical staining for SMI-31 was performed as described previously (Budde et al., 2007). After accepting antigen retrieved with the use of citrate antigen retrieval solution (P0081; Beyotime), the tissue sections were treated with 3% hydrogen peroxide to inactivate endogenous peroxidase. The sections were then incubated in 1:20 goat serum for 30 min, rinsed, and incubated overnight at 4°C with a 1:200 dilution of the primary antibody SMI-31. After three washes in PBS, the sections were incubated for 90 min with biotinylated goat anti-mice immunoglobulin G antibody (SP-9002; Zhongshan Golden Bridge Biotechnology). After another three PBS washes, the brain sections were incubated with avidin biotinylated horseradish peroxidase (SP-9002; Zhongshan Golden Bridge Biotechnology) for 90 min. The brain sections were rewashed three times in PBS and then incubated with diaminobenzidine and hydrogen peroxide (ZLI-9018; Zhongshan Golden Bridge Biotechnology). The nucleus was stained with hematoxylin. The sections were then rinsed in water for 10 min, dehydrated, and covered with a coverslip for microphotography.

### LFB Staining

For LFB staining, 5-μm-thick brain sections were deparaffinized with xylene and immersed first in 100% ethanol and then in 95% ethanol. They were incubated in 0.1% LFB solution for 24 h at room temperature. The next day, the sections were washed in distilled water for 3 min. Differentiation was accomplished by rinsing the sections in 0.05% lithium carbonate and then in 70% ethanol. The LFB-stained sections were then counterstained with cresyl violet.

### Data and Statistical Analysis

**Figure 1:**
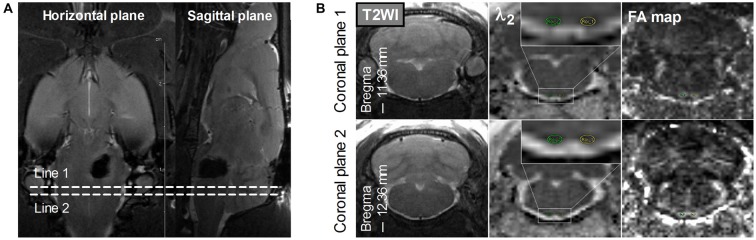
**Definition of the regions of interest (ROIs) of the bilateral pyramidal tracts (PYs). (A)** The definition of the PY (bregma, −11.36 mm to −12.36 mm) caudal to the hematoma on the horizontal and sagittal plane. Line 1 and Line 2 perpendicular to the horizontal and sagittal planes represent two planes that are located at –11.36 mm and –12.36 mm of bregma, respectively. **(B)** The definition of the bilateral PYs on two coronal planes. The images in the left column are coronal T2-weighted images. The images in the middle column are coronal λ_2_-weighted images that show the definition of the ROIs of the bilateral PYs (encircled in green and yellow and enlarged in the insets). The images in the right column are the corresponding Fractional anisotropy (FA) maps.

Hematoma volume at 2–4 h after surgery was measured on T2*-weighted images by an observer who was blinded to the rat groups. The observer outlined the regions of hypointensity that represented hemorrhage and iron accumulation on all slices, and these regions were then multiplied by section thickness (1.0 mm; Chen et al., 2011). FA, λ_1_, λ_2_, and λ_3_ maps were generated from DTI results on a local workstation. Regions of interest (ROIs) of the bilateral PYs were defined on these maps by referring to the standard rat brain atlas (Paxinos and Watson, 2007). To avoid the adverse effects of perihematomal edema and inflammation when assessing the DTI parameters (Alexander et al., 2007), two coronal planes caudal to the hematoma were selected for the ROI definition of the bilateral PY, as shown in Figure 1.

High-power images (×400 magnification) of the ipsilateral and contralateral PYs of each rat were taken separately. For the analysis of LFB and SMI-31 staining, two ROIs were selected randomly from the ipsilateral and contralateral PYs of each rat. The ROIs were binarized, and the area covered by LFB or SMI-31 staining was calculated as a percentage of the total area (Kalakh and Mouihate, 2015).

Statistical analysis was performed with the use of SPSS for Windows Version 20 (SPSS Inc., Chicago, IL, USA). Quantitative data were tested for normal distribution by using the Kolmogorov-Smirnov test. DTI parameter changes in the contralateral and ipsilateral PYs were analyzed with repeated measures analyses of variance (rmANOVA) and *post hoc* test for comparison across different time points, with Greenhouse-Geisser corrections applied when Mauchley’s test indicated that the assumption of sphericity had been violated. Levene’s test for equality of variance. Mann-Whitney *U* tests were used to compare the behavioral and histologic data. A *P* value of less than 0.05 (two tailed) was considered to indicate a significant difference for all of the statistical procedures.

## Results

### Bilateral PY Degeneration of Rats with BSH

**Figure 2:**
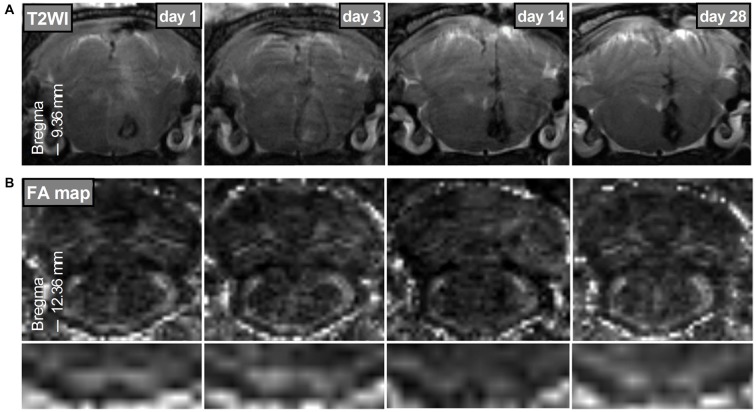
**Bilateral PYs degeneration after brain stem hemorrhage (BSH). (A)** T2-weighted images of the hematoma on days 1, 3, 14, and 28 after BSH. **(B)** FA maps of the bilateral PYs caudal to the hematoma (–12.36 mm of bregma) on days 1, 3, 14, and 28 after BSH. The bottom images are the enlarged FA maps of the bilateral PYs.

The mean hematoma volume estimated from multislice T2*-weighted images at 2–4 h after surgery was 22.71 ± 3.41 μl. The bilateral PYs adjacent to the hematoma were subjected to compressive deformation (Figure 2A). There was no apparent hematoma or perihematomal edema and no compressive deformation of the bilateral PYs in the two coronal planes caudal to the hematoma (see Figure 1B, left column). However, remarkable degeneration was found in bilateral PYs that were caudal to the hematoma, as shown in the FA maps (Figure 2B).

### Evaluation of Diffusion Tensor Imaging Parameters in the Bilateral PYs of Rats with BSH

As compared with the normal control group, the ipsilateral PY of rats with BSH displayed a significant decrease in their FA values from D3 to D28 after surgery (Table 1). The FA value in the contralateral PY decreased significantly from D7 to D28 after surgery. The ipsilateral PY of the rats with BSH showed a dramatic increase in MD from D3 to D28 after surgery. The MD in the contralateral PY showed a dramatic increase on D3 after surgery. The λ_⊥_ in the ipsilateral PY of the rats with BSH increased dramatically from D1 to D28 after surgery. It is noteworthy that the λ_⊥_ value in the ipsilateral PY measured on D28 decreased significantly as compared with that measured on D14 (*P* = 0.012). The λ_⊥_ in the contralateral PY of the rats with BSH increased significantly on D3 and D14 but returned to normal on D28. As compared with normal controls, the ipsilateral and contralateral PYs of these rats showed a decrease in λ_∥_ from D1 to D28. However, this decrease did not achieve significance. No significant differences in the DTI parameters of the PYs at different time points and between the contralateral and ipsilateral sides were found in the sham group (Table 2).

**Table 1:** **Evolution of diffusion tensor imaging (DTI) parameters in the bilateral pyramidal tracts (PYs) of rats after brain stem hemorrhage (BSH)**.

		BSH rats (*n* = 6)				
Diffusion parameters	Normal controls (*n* = 8)	Day 1	Day 3	Day 7	Day 14	Day 28	*F*	*P*
FA_ips_	0.56 ± 0.007	0.48 ± 0.032	0.40 ± 0.013***	0.39 ± 0.022**	0.37 ± 0.016***	0.41 ± 0.022**	268.96	0.000
FA_con_	0.57 ± 0.005	0.55 ± 0.031	0.50 ± 0.022	0.49 ± 0.012*	0.46 ± 0.006***	0.49 ± 0.023*	50.84	0.000
MD_ips_	0.87 ± 0.017	0.94 ± 0.032	0.99 ± 0.016**	1.01 ± 0.043*	1.00 ± 0.039*	0.97 ± 0.030*	60.44	0.000
MD_con_	0.89 ± 0.029	1.07 ± 0.083	1.05 ± 0.039*	1.01 ± 0.046	0.99 ± 0.036	0.93 ± 0.038	16.98	0.002
λ_∥ips_	1.52 ± 0.027	1.62 ± 0.086	1.55 ± 0.056	1.52 ± 0.036	1.48 ± 0.044	1.42 ± 0.035	3.42	0.094
λ_∥con_	1.60 ± 0.065	1.57 ± 0.059	1.50 ± 0.044	1.52 ± 0.087	1.49 ± 0.055	1.47 ± 0.053	0.41	0.536
λ_⊥ips_	0.57 ± 0.009	0.71 ± 0.045*	0.82 ± 0.031**	0.82 ± 0.042**	0.85 ± 0.015***	0.75 ± 0.036**	154.99	0.000
λ_⊥con_	0.57 ± 0.018	0.62 ± 0.036	0.68 ± 0.035*	0.68 ± 0.030	0.73 ± 0.024**	0.65 ± 0.032	16.99	0.000

Data are shown as mean ± standard error of the mean; *P < 0.05, **P < 0.01 and ***P < 0.001 vs. normal controls; P values indicate overall significance between different time points. FA, fractional anisotropy; MD, mean diffusivity; λ_∥_, axial diffusivity; λ_⊥_, radial diffusivity; ips, ipsilateral; con, contralateral.

**Table 2:** **Evolution of DTI parameters in the bilateral PYs of the rats in the sham group**.

		Sham rats (*n*) = 6)				
Diffusion parameters	Normal controls (*n* = 8)	Day 1	Day 3	Day 7	Day 14	Day 28	*F*	*P*
FA_ips_	0.56 ± 0.007	0.57 ± 0.011	0.56 ± 0.004	0.56 ± 0.004	0.57 ± 0.004	0.56 ± 0.005	0.33	0.712
FA_con_	0.57 ± 0.005	0.56 ± 0.003	0.57 ± 0.009	0.57 ± 0.004	0.57 ± 0.005	0.56 ± 0.005	0.40	0.845
MD_ips_	0.87 ± 0.017	0.84 ± 0.050	0.89 ± 0.021	0.84 ± 0.026	0.88 ± 0.014	0.87 ± 0.016	0.46	0.804
MD_con_	0.89 ± 0.029	0.86 ± 0.045	0.84 ± 0.060	0.87 ± 0.023	0.89 ± 0.024	0.87 ± 0.019	0.46	0.579
λ_∥ips_	1.52 ± 0.027	1.44 ± 0.096	1.48 ± 0.043	1.44 ± 0.049	1.48 ± 0.028	1.49 ± 0.032	0.45	0.813
λ_∥con_	1.60 ± 0.065	1.47 ± 0.074	1.42 ± 0.111	1.50 ± 0.050	1.52 ± 0.048	1.49 ± 0.036	0.42	0.616
λ_⊥ips_	0.57 ± 0.009	0.55 ± 0.029	0.57 ± 0.012	0.55 ± 0.012	0.56 ± 0.007	0.56 ± 0.009	0.59	0.707
λ_⊥con_	0.57 ± 0.018	0.56 ± 0.029	0.54 ± 0.033	0.57 ± 0.011	0.57 ± 0.014	0.56 ± 0.011	0.41	0.617

*Data are shown as mean ± standard error of the mean; P values indicate overall significance between different time points. FA, fractional anisotropy; MD, mean diffusivity; λ_∥_, axial diffusivity; λ_⊥_, radial diffusivity; ips, ipsilateral; con, contralateral*.

### Axon and Myelin Loss in the Bilateral PYs of Rats with BSH

**Figure 3:**
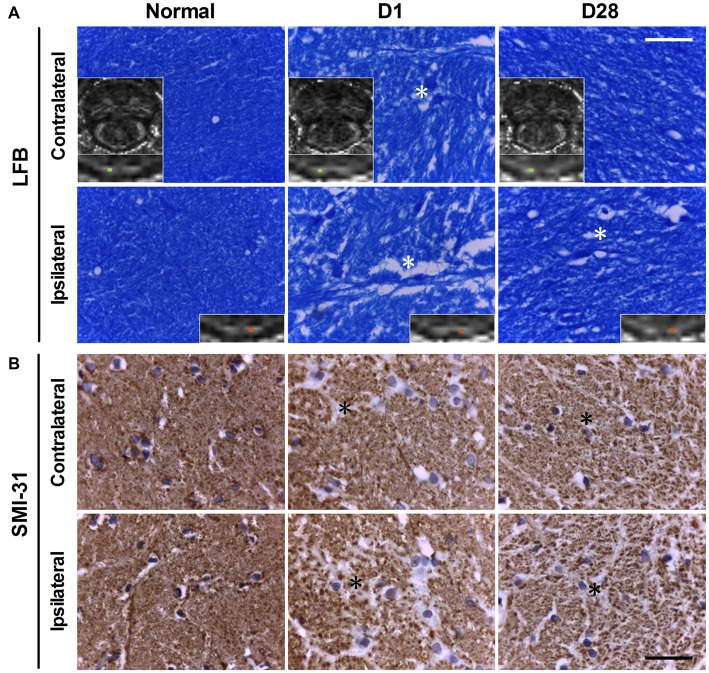
**Luxol fast blue (LFB; A) and SMI-31 (B) staining of the bilatertal PYs of rats with BSH and normal controls.** The blue color in panel **(A)** represents myelin. The insets are corresponding FA maps with enlarged views of the bilateral PYs, with small cyan boxes denoting the areas that correspond to the histologic images. White asterisks indicate areas of myelin loss. The brown dots in panel **(B)** represent cross views of axons. Black asterisks indicate areas of axon loss. Scale bar = 30 μm.

The myelin and axons of the bilateral PYs were stained by LFB and SMI-31, respectively. In normal controls, LFB staining showed that the myelin sheath of the PY was intact, regular, and tight, with a lamellar structure. The SMI-31 staining showed intact, regular, and tightly arranged axons. As compared with normal controls, rats with BSH demonstrated apparent myelin loss in the bilateral PYs from D1 to D28 after surgery (as indicated by the white asterisks in Figure 3A). Progressively dramatic axon loss in the bilateral PYs was also detected in the rats with BSH (as indicated by the black asterisks in Figure 3B). No apparent histologic changes were observed in the sham control group.

**Figure 4:**
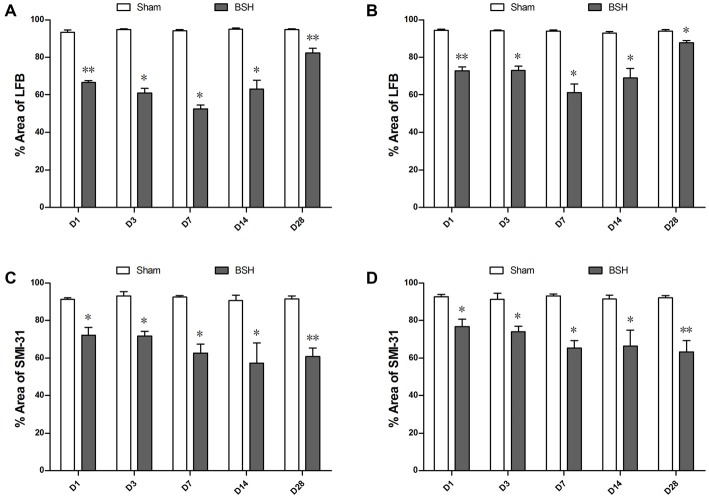
**Myelin and axon loss in the bilateral PYs of rats with BSH. (A)** Percentage area covered by LFB staining in the ipsilateral PY of rats with BSH and sham controls. **(B)** Percentage area covered by LFB staining in the contralateral PY of rats with BSH and sham controls. **(C)** Percentage area covered by SMI-31 staining in the ipsilateral PY of rats with BSH and sham controls. **(D)** Percentage area covered by SMI-31 staining in the contralateral PY of rats with BSH and sham controls. Data are shown as mean ± standard error of the mean. *P < 0.05 and **P < 0.01 vs. sham controls, Mann-Whitney *U* tests.

As compared with sham controls, the ipsilateral PY of rats with BSH showed statistically significant myelin loss on D1, D3, D7, D14, and D28 after surgery at rates of 28.70%, 35.64%, 44.19%, 33.66%, and 13.17%, respectively (Figure 4A). The myelin in the contralateral PY of the rats with BSH decreased by 22.80%, 22.49%, 34.83%, 25.57%, and 6.58%, respectively, at the same time points (Figure 4B). As compared with sham controls, the ipsilateral PY of the rats with BSH showed progressive axon loss on D1, D3, D7, D14, and D28 after surgery at rates of 22.88%, 22.94%, 32.12%, 36.86%, and 33.43%, respectively (Figure 4C). The axons in the contralateral PY of the rats with BSH decreased progressively by 17.21%, 18.81%, 29.94%, 27.47%, and 31.22%, respectively (Figure 4D).

### Motor Function Damage and Recovery of Rats with BSH

**Figure 5:**
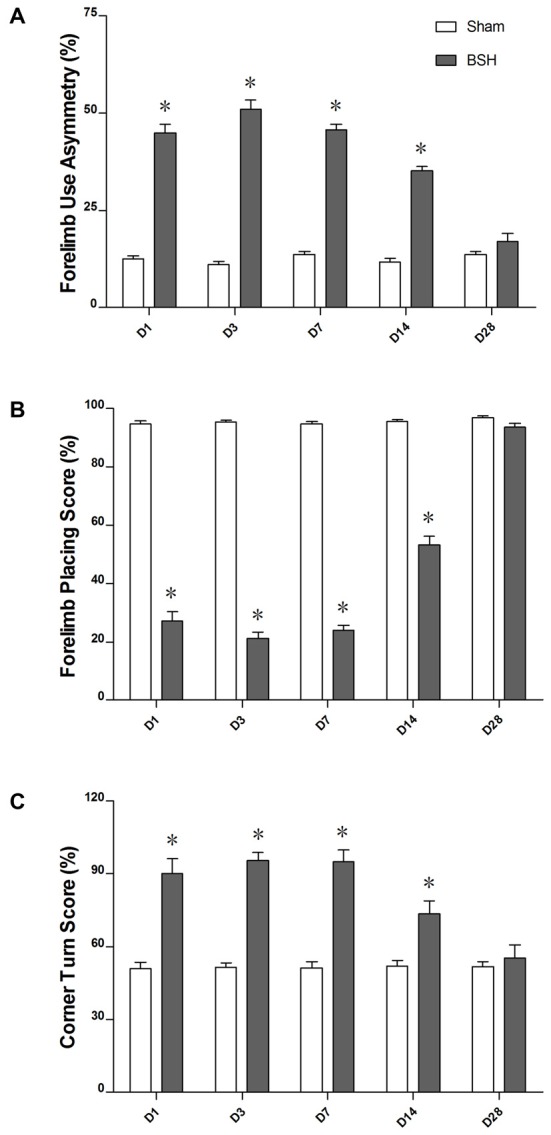
**Forelimb use asymmetry scores (A), forelimb placing scores (B), and corner turn scores (C) of rats with BSH and sham controls.** Data are shown as mean ± standard error of the mean.*P < 0.01 vs. sham controls, Mann-Whitney *U* tests.

As compared with sham controls, the rats with BSH demonstrated noticeable forelimb use asymmetry from D1 to D14 after surgery; however, the deficit disappeared and the motor function returned to normal by D28 (Figure 5A). The rats with BSH showed significant forelimb placing deficits as compared with sham controls from D1 to D14 after surgery; these deficits were most severe on D3 and D7 but returned to normal by D28 (Figure 5B). For the corner turn test, there was a significant increase in the percentage of left (contralateral) turns of the rats with BSH as compared with sham controls from D1 to D14 after surgery, but the corner turn scores of the rats with BSH had returned to normal by D28 (Figure 5C).

## Discussion

To our knowledge, this study is the first of its kind to evaluate the dynamic changes of the bilateral PYs of rats with BSH by using DTI. As compared with normal controls, the bilateral PYs of these rats showed an early decrease and a late increase in FA and an early increase and a late decrease in MD. A progressive decrease in λ_∥_ with dramatic axon loss from D1 to D28 after BSH was found bilaterally. The bilateral PYs of rats with BSH showed an early increase and a late decrease in λ_⊥_. Early myelin injury and late repair were also detected pathologically in the bilateral PYs of rats with BSH. Thus, the early motor function deficits of rats with BSH began to improve by D14, and they had almost completely disappeared by D28.

### Dynamic Changes in the Diffusion Indices of the Degenerated PYs After BSH

Neuronal fiber tract degeneration has been demonstrated to occur after hemorrhagic stroke (Yeo et al., 2012; Fan et al., 2013; Ma et al., 2014). In most of the ICH studies, the hematoma was mostly supratentorial. Few studies have focused on infratentorial hemorrhage, especially with BSH or pontine hemorrhage, likely because of the difficulties associated with large-scale clinical data collection and the establishment of an animal model. To our knowledge, no study has investigated fiber tract degeneration after BSH using DTI techniques. In addition, although it has been determined that the PY degeneration occurs several hours to days after the ICH (Fan et al., 2013), questions that address how early DTI can detect the PY changes and to what extent DTI can provide accurate and detailed information about PY degeneration and repair after BSH have not yet been answered. In response, we have developed a novel BSH model by injecting autologous blood into the rat brain stem. On the basis of this model, we evaluated the bilateral PY changes using DTI and histopathology on D1, D3, D7, D14, and D28 after blood injection.

The FA of the ipsilateral PY decreased monotonously from D3 to D14 and increased but was not completely recovered by D28, with MD increasing from D3 to D28. These changes were consistent with previous experimental and clinical ICH studies (Yoshioka et al., 2008; Kusano et al., 2009; Fan et al., 2013). As described previously, hematoma may lead to nerve fiber degeneration due to mechanical effects, perihematomal ischemia, and secondary injury as a result of neuroinflammation and the release of neurotoxic materials (Xi et al., 2006; Keep et al., 2012; Chaudhary et al., 2015). A series of such attacks could lead to neuron death, axon degradation, and myelin injury. In the current study, the ipsilateral PY of BSH rats showed a monotonous decrease of λ_∥_ and an increase in λ_⊥_ from D1 to D28 after surgery, which were in line with previous studies (Yu et al., 2009; Fan et al., 2013). Progressive axon and myelin loss from D1 to D28 detected by histologic and pathologic tests could partly explain the changes in λ_∥_ and λ_⊥_ accordingly (Budde et al., 2007; Wang et al., 2008). The decrease in λ_∥_ can be ascribed to the fragmentation of the axons, which creates barriers to the longitudinal displacement of water molecules; the increase in λ_⊥_ corresponds to the degradation of the myelin sheath, which causes water molecules to become more mobile perpendicular to the axons (Yu et al., 2009). The increased λ_⊥_ and the decreased λ_∥_ led to the decrease in FA and the increase in MD (Sidaros et al., 2008; Yu et al., 2009). In this study, the increase in FA in the ipsilateral PY on D28 may be ascribed to the higher proportion of the decrease in λ_⊥_ as compared with the decrease in λ_∥_.

Our results showed that the increase in λ_⊥_ and the decrease in FA in the contralateral PY were less obvious than the similar changes seen in the ipsilateral PY. As shown in Figure 2A, the contralateral PY was not unaffected by the hematoma, although its involvement was not so severe. In addition, our histologic and pathologic staining results showed that there were crossed fibers in the bilateral PY. To some extent, the crossed fibers from the contralateral PY may change the diffusion tensor parameters (Pierpaoli et al., 2001). Further studies should be performed to elucidate if there are other mechanisms involved.

### The Possible Mechanism of Motor Function Recovery After BSH and its Correlation with PY Integrity

Previous studies have demonstrated that the changes in the DTI parameters in the corticospinal tract (CST) correlate with clinical signs and can thus help to predict functional outcomes (Thomalla et al., 2004; Kusano et al., 2009; DeVetten et al., 2010; Zhang et al., 2015). In the current study, the rats with BSH demonstrated severe motor deficits and delayed recovery that reached their maximums and plateaued from D3 to D7; these deficits showed improvement from D14 on and were normalized by D28. It is interesting to note that the motor function began to recover on D14; the DTI parameters did not recover and in fact remained at their lowest levels. This may be ascribed to the perilesional reorganization that occurs after a stroke insult (Jang, 2011). A recent clinical pontine infarct study demonstrated the regeneration and reorganization of the motor pathways (Zhang et al., 2015). In that study, fibers arose from the contralateral CST and crossed over to the ipsilateral side and down the edge of the infarct during the recovery of motor function in some patients with severe CST damage. Some of these fibers ran along the conventional anatomic route of the CST, whereas others merged with the pontocerebellar fibers, which are particularly important for motor function (Lu et al., 2011). The reorganization of the motor pathways in regions other than the PY contributed partly to the degree of motor recovery. However, the bilateral PY degenerated progressively. This motor recovery theory may also help to explain the conflict between progressive axon injury and neurologic recovery. Additional in-depth studies should be performed to elucidate the mechanism of motor function recovery after BSH.

## Conclusions

DTI revealed dynamic structural changes in both the contralateral and ipsilateral PYs after experimental BSH. The evolution of the bilateral parameters has been demonstrated by histologic and pathologic staining and correlated with motor function changes. Therefore, quantitative multiparametric DTI can track the structural changes of the bilateral PYs and thus may serve as a noninvasive tool to predict the prognoses of patients with BSH.

## Author Contributions

R-ZZ: carried out study design, animal model creation, MR scans, MR data analysis and interpretation, manuscript writing and revision; C-YT: carried out study design, animal model creation, behavioral data analysis and interpretation, manuscript writing and revision; WC: carried out MR sequence refining and setup, MR data analyses and figure production; C-HW: carried out MR scans, MR data acquisition and analyses, behavioral tests; YH: carried out histological examination, histological data analysis, interpretation and figure production; LS: carried out MR scans, MR data acquisition, behavioral tests; BZ: carried out MR data collection and animal care; Y-SC: carried out MR data compilation and animal care; Z-QX: carried out histological examination, histological data collection and analysis; LW: carried out MR sequence refining and setup; HF: carried out manuscript revision; T-HW: carried out manuscript revision; JZ: carried out MR sequence refining and manuscript revision; CY: carried out conception and design of the work, manuscript revision; F-BG: carried out conception and design of the work, manuscript revision.

## Funding

This study was partly supported by the State Key Program of National Natural Science Foundation of China (No. 81130027); the National Key Basic Research Program of China (No. 2014CB541600); the International Cooperation and Exchange Projects of the National Natural Science Foundation of China (No. 81520108014); the Joint Research Fund for Overseas Chinese, Hong Kong and Macao Young Scholars (No. 81528009), and the National Key Technology R&D Program for the 12th Five-year Plan of China (No. 2011BAI08B05).

## Conflict of Interest Statement

The authors declare that the research was conducted in the absence of any commercial or financial relationships that could be construed as a potential conflict of interest.

## References

[B1] Alexander A. L. Lee J. E. Lazar M. Field A. S. (2007). Diffusion tensor imaging of the brain. Neurotherapeutics 4, 316–329. 10.1016/j.nurt.2007.05.011 17599699 PMC2041910

[B2] Budde M. D. Kim J. H. Liang H. F. Schmidt R. E. Russell J. H. Cross A. H.. (2007). Toward accurate diagnosis of white matter pathology using diffusion tensor imaging. Magn. Reson. Med. 57, 688–695. 10.1002/mrm.21200 17390365

[B3] Chaudhary N. Pandey A. S. Gemmete J. J. Hua Y. Huang Y. Gu Y.. (2015). Diffusion tensor imaging in hemorrhagic stroke. Exp. Neurol. 272, 88–96. 10.1016/j.expneurol.2015.05.011 26015333 PMC4631675

[B4] Chen Z. Gao C. Hua Y. Keep R. F. Muraszko K. Xi G. (2011). Role of iron in brain injury after intraventricular hemorrhage. Stroke 42, 465–470. 10.1161/STROKEAHA.110.602755 21164132 PMC3078056

[B5] DeVetten G. Coutts S. B. Hill M. D. Goyal M. Eesa M. O’Brien B.. (2010). Acute corticospinal tract Wallerian degeneration is associated with stroke outcome. Stroke 41, 751–756. 10.1161/STROKEAHA.109.573287 20203322

[B6] Fan S.-J. Lee F. Y. Cheung M. M. Ding A. Y. Yang J. Ma S. J.. (2013). Bilateral substantia nigra and pyramidal tract changes following experimental intracerebral hemorrhage: an MR diffusion tensor imaging study. NMR Biomed. 26, 1089–1095. 10.1002/nbm.2922 23417762

[B7] Hua Y. Schallert T. Keep R. F. Wu J. Hoff J. T. Xi G. (2002). Behavioral tests after intracerebral hemorrhage in the rat. Stroke 33, 2478–2484. 10.1161/01.str.0000032302.91894.0f 12364741

[B8] Jang S. H. (2011). A review of diffusion tensor imaging studies on motor recovery mechanisms in stroke patients. NeuroRehabilitation 28, 345–352. 10.3233/NRE-2011-0662 21725167

[B9] Kalakh S. Mouihate A. (2015). The promyelinating properties of androstenediol in gliotoxin-induced demyelination in rat corpus callosum. Neuropathol. Appl. Neurobiol. 41, 964–982. 10.1111/nan.12237 25786683

[B10] Keep R. F. Hua Y. Xi G. (2012). Intracerebral haemorrhage: mechanisms of injury and therapeutic targets. Lancet Neurol. 11, 720–731. 10.1016/s1474-4422(12)70104-7 22698888 PMC3884550

[B11] Kumar R. Woo M. A. Macey P. M. Fonarow G. C. Hamilton M. A. Harper R. M. (2011). Brain axonal and myelin evaluation in heart failure. J. Neurol. Sci. 307, 106–113. 10.1016/j.jns.2011.04.028 21612797 PMC3150745

[B12] Kusano Y. Seguchi T. Horiuchi T. Kakizawa Y. Kobayashi T. Tanaka Y.. (2009). Prediction of functional outcome in acute cerebral hemorrhage using diffusion tensor imaging at 3T: a prospective study. AJNR Am. J. Neuroradiol. 30, 1561–1565. 10.3174/ajnr.A1639 19556354 PMC7051627

[B13] Kuzu Y. Inoue T. Kanbara Y. Nishimoto H. Fujiwara S. Ogasawara K.. (2012). Prediction of motor function outcome after intracerebral hemorrhage using fractional anisotropy calculated from diffusion tensor imaging. Cerebrovasc. Dis. 33, 566–573. 10.1159/000338904 22688137

[B14] Lekic T. Rolland W. Manaenko A. Krafft P. R. Kamper J. E. Suzuki H.. (2013). Evaluation of the hematoma consequences, neurobehavioral profiles and histopathology in a rat model of pontine hemorrhage. J. Neurosurg. 118, 465–477. 10.3171/2012.10.JNS111836 23198805 PMC3569075

[B15] Lu J. Liu H. Zhang M. Wang D. Cao Y. Ma Q.. (2011). Focal pontine lesions provide evidence that intrinsic functional connectivity reflects polysynaptic anatomical pathways. J. Neurosci. 31, 15065–15071. 10.1523/JNEUROSCI.2364-11.2011 22016540 PMC3397237

[B16] Ma C. Liu A. Li Z. Zhou X. Zhou S. (2014). Longitudinal study of diffusion tensor imaging properties of affected cortical spinal tracts in acute and chronic hemorrhagic stroke. J. Clin. Neurosci. 21, 1388–1392. 10.1016/j.jocn.2013.11.032 24746110

[B17] Meguro T. Kuwahara K. Tomita Y. Okuma Y. Tanabe T. Muraoka K.. (2015). Primary pontine hemorrhage in the acute stage: clinical features and a proposed new simple scoring system. J. Stroke Cerebrovasc. Dis. 24, 860–865. 10.1016/j.jstrokecerebrovasdis.2014.12.006 25724243

[B18] Paxinos G. Watson C. (2007). The Rat Brain in Stereotaxic Coordinates. San Diego: Elsevier Academic Press.

[B19] Pierpaoli C. Barnett A. Pajevic S. Chen R. Penix L. R. Virta A.. (2001). Water diffusion changes in Wallerian degeneration and their dependence on white matter architecture. Neuroimage 13, 1174–1185. 10.1006/nimg.2001.0765 11352623

[B20] Qureshi A. I. Tuhrim S. Broderick J. P. Batjer H. H. Hondo H. Hanley D. F. (2001). Spontaneous intracerebral hemorrhage. N. Engl. J. Med. 344, 1450–1460. 10.1056/NEJM200105103441907 11346811

[B21] Sidaros A. Engberg A. W. Sidaros K. Liptrot M. G. Herning M. Petersen P.. (2008). Diffusion tensor imaging during recovery from severe traumatic brain injury and relation to clinical outcome: a longitudinal study. Brain 131, 559–572. 10.1093/brain/awm294 18083753

[B22] Song S. K. Sun S. W. Ramsbottom M. J. Chang C. Russell J. Cross A. H. (2002). Dysmyelination revealed through MRI as increased radial (but unchanged axial) diffusion of water. Neuroimage 17, 1429–1436. 10.1006/nimg.2002.1267 12414282

[B23] Takeuchi S. Suzuki G. Takasato Y. Masaoka H. Hayakawa T. Otani N.. (2013). Prognostic factors in patients with primary brainstem hemorrhage. Clin. Neurol. Neurosurg. 115, 732–735. 10.1016/j.clineuro.2012.08.022 22944466

[B24] Thomalla G. Glauche V. Koch M. A. Beaulieu C. Weiller C. Rother J. (2004). Diffusion tensor imaging detects early Wallerian degeneration of the pyramidal tract after ischemic stroke. Neuroimage 22, 1767–1774. 10.1016/j.neuroimage.2004.03.041 15275932

[B25] van der Eerden A. W. Khalilzadeh O. Perlbarg V. Dinkel J. Sanchez P. Vos P. E.. (2014). White matter changes in comatose survivors of anoxic ischemic encephalopathy and traumatic brain injury: comparative diffusion-tensor imaging study. Radiology 270, 506–516. 10.1148/radiol.13122720 24471392

[B26] Wang S. Wu E. X. Tam C. N. Lau H. F. Cheung P. T. Khong P. L. (2008). Characterization of white matter injury in a hypoxic-ischemic neonatal rat model by diffusion tensor MRI. Stroke 39, 2348–2353. 10.1161/STROKEAHA.107.509927 18535275

[B27] Wijdicks E. F. St Louis E. (1997). Clinical profiles predictive of outcome in pontine hemorrhage. Neurology 49, 1342–1346. 10.1212/wnl.49.5.1342 9371919

[B28] Xi G. Keep R. F. Hoff J. T. (2006). Mechanisms of brain injury after intracerebral haemorrhage. Lancet Neurol. 5, 53–63. 10.1016/s1474-4422(05)70283-0 16361023

[B29] Yeo S. S. Choi B. Y. Chang C. H. Kim S. H. Jung Y. J. Jang S. H. (2012). Evidence of corticospinal tract injury at midbrain in patients with subarachnoid hemorrhage. Stroke 43, 2239–2241. 10.1161/STROKEAHA.112.661116 22700530

[B30] Yoshioka H. Horikoshi T. Aoki S. Hori M. Ishigame K. Uchida M.. (2008). Diffusion tensor tractography predicts motor functional outcome in patients with spontaneous intracerebral hemorrhage. Neurosurgery 62, 97–103; discussion 103. 10.1227/01.NEU.0000311066.03121.b8 18300896

[B31] Yu C. Zhu C. Zhang Y. Chen H. Qin W. Wang M.. (2009). A longitudinal diffusion tensor imaging study on Wallerian degeneration of corticospinal tract after motor pathway stroke. Neuroimage 47, 451–458. 10.1016/j.neuroimage.2009.04.066 19409500

[B32] Zhang M. Lin Q. Lu J. Rong D. Zhao Z. Ma Q.. (2015). Pontine infarction: diffusion-tensor imaging of motor pathways-a longitudinal study. Radiology 274, 841–850. 10.1148/radiol.14140373 25356962 PMC4556232

